# Difficulté diagnostique d'une tuberculose laryngée isolée chez une femme diabétique

**DOI:** 10.11604/pamj.2015.21.106.6361

**Published:** 2015-06-09

**Authors:** Madiha Mahfoudhi, Khaled Khamassi, Sami Turki, Adel Kheder

**Affiliations:** 1 Service de Médecine Interne A, Hôpital Charles Nicolle, Tunis, Tunisie; 2 Service d'ORL, Hôpital Charles Nicolle, Tunis, Tunisie

**Keywords:** Mycobacterium tuberculosis, larynx, nécrose caséeuse, diabète, Mycobacterium tuberculosis, larynx, caseous necrosis, diabetes

## Abstract

La tuberculose laryngée isolée est rare. Elle peut mimer une tumeur maligne retardant le diagnostic et aggravant le pronostic. Le terrain diabétique favorise la survenue des infections notamment la tuberculose dans sa forme sévère. Nous rapportons l'observation d'une patiente diabétique de type 2, âgée de 51 ans, hospitalisée pour une fièvre prolongée, une dysphonie et un diabète déséquilibré. Les hémocultures étaient négatives. L'examen laryngoscopique a confirmé un aspect érythémateux et épais des deux cordes, une lésion ulcérée peu profonde de l'hémilarynx gauche et une végétation érythémateuse de 5 mm de diamètre. L'examen histologique a révélé des granulomes épithéloïodes et giganto-cellulaires avec une nécrose caséeuse évocateurs de la tuberculose. La culture sur milieu de lobstein de tissus biopsiés à partir des cordes vocales a été positive. Aucun autre foyer tuberculeux n'a été retrouvé. Le traitement par une combinaison de l'isoniazide, la rifampicine, l’éthambutol, la pyrazinamide a permis une résolution de symptômes.

## Introduction

La tuberculose est une infection due au *Mycobacterium tuberculosis*. Elle survient chez les sujets immunocompétents mais elle est plus fréquents chez les immunodéprimés notamment les diabétiques [1]. La forme pulmonaire est la plus fréquente. La localisation laryngée est rare. Lorsque manifestations laryngés sont associés à des signes pulmonaires, le diagnostic de la tuberculose est facile. Cependant, la tuberculose laryngée primaire est rare et peut se manifester par une tumeur induisant un temps de retard de diagnostic. Les examens cliniques et endoscopiques n'ont pas de spécificité dans le cas de la tuberculose laryngée. Seuls les résultats histologiques typiques ou les résultats bactériologiques permettent de confirmer l'origine tuberculeuse de la symptomatologie laryngée. Le but de cette étude est d'insister sur la difficulté diagnostique de la tuberculose laryngée pouvant simuler une néoplasie, une vascularite, une sarcoïdose, un lymphome ou une autre infection.

## Patient et observation

Une femme âgée de 51 ans, non tabagique, suivi pour un diabète de type 2 depuis 2 ans, sous antidiabétiques oraux, hospitalisée pour une fièvre prolongée évoluant depuis un mois, des sueurs nocturnes. Elle avait une anorexie et un amaigrissement chiffré à 7 Kg en deux mois. Il n'y avait pas de notion d'antécédents personnels de tuberculose ni de contage tuberculeux. Elle a été vaccinée par le vaccin BCG et n'avait pas reçu de traitement corticoïde ou immunosuppresseur. Par ailleurs, elle se plaignait d'une dysphonie récente. L'examen physique a révélé une fièvre de 38.5 ^°^C. Il n'y avait pas d'adénopathie, ni d'hépato-splénomégalie. Les examens cardiaques, respiratoires, neurologiques, musculaires, ophtalmologiques et ostéo-articulaire étaient sans anomalies. Plusieurs séries d'hémocultures réalisées en urgence étaient négatives. L'examen biologique a révélé un syndrome inflammatoire avec une vitesse de sédimentation à 70 mm, une C réactive protéine à 35 mg/l et une hypergammaglobulinémie polyclonale à 18 g/l. L’étude de la numération formule sanguine a objectivé une anémie hypochrome microcytaire inflammatoire (hémoglobine: 10,5g / dl), des globules blancs à 4500/mm^3^ et un taux normal des plaquettes de 450000/mm^3^. Il n'y avait pas de stigmates biologiques de malabsorption. Les fonctions rénales et hépatiques étaient normales. Par ailleurs, son diabète était déséquilibré malgré un bon régime et augmentation des doses d'anti-diabétiques oraux. En outre, les examens bactériologiques des crachats et de l'urine à la recherche de *Mycobacterium tuberculosis* étaient négatifs. Par ailleurs, le bilan immunologique (ANA, ANCA) était négatif.

La laryngoscopie a confirmé un aspect érythémateusx et épais des deux cordes vocales et une ulcération peu profonde de 10 mm de grand axe au niveau de l'hémilarynx gauche et une végétation érythémateuse de 5 mm de diamètre. Aucune paralysie ou parésie des cordes vocales a été mentionné. L'examen histologique des biopsies réalisées à partir des cordes vocales, de l'ulcération et la végétation a trouvé des infiltrats inflammatoires organisées sous forme de granulomes associant des lymphocytes, des cellules épithéloïde et des cellules géantes, une fibrose et une nécrose caséeuse. Aucun signes de malignité ni de vascularite n'a été objectivé. Ces résultats étaient évocateurs du diagnostic de tuberculose laryngée. La coloration de Ziehl Neelsen a révélé des bacilles acido-alcoolo-résistants. La culture de tissus laryngés lésés sur milieu de Lobstein était positive. Aucun autre foyer de tuberculose n'a été authentifié ni par l'examen physique, ni par l'imagerie (radiographie standard et tomodensitométrie). Le traitement par un traitement oral correspondant à une combinaison d'isoniazide, rifampicine, pyrazinamide et éthambutol pendant deux mois puis relai par l'association isoniazide et rifampicine pendant dix mois a permis une résolution totale de signes cliniques, biologiques et endoscopiques avec équilibration du diabète. Le patient n'a présenté aucun effet indésirable de son traitement anti-tuberculeu

## Discussion

La tuberculose est une infection à *Mycobacterium tuberculosis* dont le siège est essentiellement pulmonaire. Elle peut survenir chez les patients immunocompétents, ou le plus souvent immunodéprimés notamment les diabétiques [1]. La tuberculose peut se manifester sous une forme latente et chronique conduisant à un délabrement tissulaire irréductible voire une miliaire tuberculeuse en cas de retard diagnostique. La vaccination contre la tuberculose a permis une baisse de l'incidence de cette maladie. Cependant, certaines zones sont encore considérées comme des zones endémiques où le diagnostic de la tuberculose doit être envisagé. Ainsi, le profil vaccinal doit être vérifié surtout chez les patients immunodéprimés qui vivent dans ces zones. Par conséquent, les caractéristiques cliniques de la tuberculose ont été modifiées en raison de la disponibilité des médicaments antituberculeux et l'augmentation de la fréquence des conditions immunodépressives. Ces conditions ont favorisé l’émergence de formes de tuberculose extra-pulmonaire impliquant le rachis, les muscles, les méninges, l'hypophyse, la thyroïde, le système digestif, les organes hématopoïétiques, le larynx, les sinus, le nez… Plusieurs études ont montré qu'une tuberculose pulmonaire ou extra-pulmonaire se manifeste sous formes de tableaux sévères et de mauvais pronostic en cas de diabète et surtout en cas de retard diagnostique [2]. D'autres études ont prouvé la difficulté d’équilibration du diabète chez des patients tuberculeux tant que la tuberculose n'a pas été jugulée [2]. Une étude réalisée à Guyana a colligé 100 diabétiques dont 90 cas de tuberculose pulmonaire et 10 cas de tuberculose extra-pulmonaire [3]. Une autre étude a montré que l'incidence de la tuberculose extra-pulmonaire notamment péritonéale était plus élevée chez les patients diabétiques que les patients non diabétiques [4]. Les localisations tuberculeuses laryngées isolées sont rares. En effet, la tuberculose laryngée est toujours associée à l'atteinte pulmonaire et survient généralement chez des patients sans vaccination par le BCG ou en cas de déficit immunitaire [5, 6]. La tuberculose laryngée primaire peut se manifester par un enrouement chronique ou une dysphonie dans un contexte fébrile. El Ayoubi F et al ont étudié dix cas de tuberculose laryngée primaire. Dans cette série, les patients étaient des fumeurs de sexe masculin. Ils n'avaient pas de signes cliniques ou endoscopiques spécifiques. Les tableaux cliniques de ces patients simulaient ceux du cancer du larynx. Seuls les examens bactériologiques et histologiques permettent la confirmation diagnostique [5]. Une tumeur maligne, un lymphome ou une vascularite telle que la maladie de Wegener et une sarcoïdose sont considérés les principaux diagnostics différentiels d'une tuberculose laryngée. Les résultats endoscopiques peuvent montrer des ulcérations ou des végétations. La série d’études de Hasibi M et al ont révélé que les aspects macroscopiques des lésions laryngées étaient végétantes dans 11 cas et ulcéreuse dans 14 cas [7]. La lésion du larynx est chronique, persistante et réfractaire malgré l'administration d'un traitement symptomatique.

Le premier diagnostic évoqué dans de nombreux cas publiés devant ces lésions était une tumeur maligne ou une inflammation non spécifique [7–9]. El Kettani NE et al ont rapporté le cas d'un homme souffrant d'une dysphonie et dont la laryngoscopie a montré une lésion de la corde vocale gauche et le scanner trouve un épaississement focal irrégulier de la paroi postérieure du larynx évoquant un carcinome du larynx [10]. La tuberculose laryngée est le plus souvent initialement diagnostiquée comme un cancer [11]. Une intra-dermo réaction à la tuberculine positive et un Quantiféron test positif sont en faveur de lésions tuberculeuses. La coloration de Ziehl Neelson montrant des bacilles acido- alcoolo-résistant et culture spécifique positive sur milieu de Lobstein sont de bons arguments pour retenir l'origine tuberculeuse. Les résultats histologiques peuvent être typiques, tel que présenté dans notre cas (inflammation chronique granulomateuse avec cellules géantes, présence de nécrose caséeuse et de cellules de type Langhans) confirmant ainsi le diagnostic ou atypiques (inflammation granulomatose chronique ou une inflammation chronique sans nécrose) responsables d'un retard diagnostique [7, 9]. Hasibi M et al ont rapporté 19 cas de tuberculose laryngée avec lésions histologiques typiques alors que 6 patients avaient un aspect anatomopathologique atypique (inflammation granulomateuse ou chronique sans nécrose). Le délai diagnostique par rapport au début de la symptomatologie était de 2-12 mois. Ce retard diagnostique peut aggraver le pronostic laryngé, surtout sur un terrain immunodéprimé [7]. La tuberculose laryngée primaire devrait être suspectée dans les pays endémiques, même si les résultats cliniques et endoscopiques sont atypiques [7, 8]. Seul un diagnostic précoce et un traitement spécifique permettent la résolution de tous les symptômes. La réponse au traitement antituberculeux était bonne dans notre cas et dans la plupart des publications [7, 12]. Une fois le diagnostic confirmé, un traitement antituberculeux administré chez dix patients pour une durée minimale de 6 mois a donné des résultats excellents à court terme et à long terme [5]. Cette durée a été prolongée dans notre cas à douze mois pour éviter une destruction tissulaire irréductible du larynx.

## Conclusion

Il est important de considérer la tuberculose laryngée comme une maladie infectieuse rare qui peut compliquer un diabète et qui présente des signes cliniques variables et des résultats endoscopiques polymorphes, mais pour lesquels il existe un traitement médical très efficace.

## References

[1] Feleke Y, Abdulkadir J, Aderaye G (1999). Prevalence and clinical features of tuberculosis in Ethiopian diabetic patients. East Afr Med J..

[2] Podell BK, Ackart DF, Obregon-Henao A, Eck SP, Henao-Tamayo M, Richardson M (2014). Increased severity of tuberculosis in Guinea pigs with type 2 diabetes: a model of diabetes-tuberculosis comorbidity. Am J Pathol..

[3] Alladin B, Mack S, Singh A, Singh C, Smith B, Cummings E (2011). Tuberculosis and diabetes in Guyana. Int J Infect Dis..

[4] Weng SF, Hsu CH, Lirn ML, Huang CL (2009). Extrapulmonary tuberculosis: a study comparing diabetic and nondiabetic patients. Exp Clin Endocrinol Diabetes..

[5] El Ayoubi F, Chariba I, El Ayoubi A, Chariba S, Essakalli L (2014). Primary tuberculosis of the larynx. Eur Ann Otorhinolaryngol Head Neck Dis..

[6] Fsadni P, Fsadni C, Caruana Montaldo B (2014). Primary laryngeal tuberculosis: an unusual cause of hoarseness. Ear Nose Throat J..

[7] Hasibi M, Yazdani N, Asadollahi M, Sharafi M, Dehghan Manshadi SA (2013). Clinical features of laryngeal tuberculosis in Iran. Acta Med Iran..

[8] Cruz S, Ribeiro A, Trigueiros N, Rodrigues E, Rodrigues M (2014). Laryngeal tuberculosis: a diagnosis not to be overlooked. Eur Ann Otorhinolaryngol Head Neck Dis..

[9] Benwill JL, Sarria JC (2014). Laryngeal tuberculosis in the United States of America: a forgotten disease. Scand J Infect Dis..

[10] El Kettani NE, El Hassani M, Chakir N, Jiddane M (2010). Primary laryngeal tuberculosis mimicking laryngeal carcinoma: CT scan features. Indian J Radiol Imaging..

[11] Baxi S, Jha S (2010). Primary laryngeal tuberculosis--a rare entity. J Indian Med Assoc..

[12] Levian M, Chapman A, Gupta R (2014). Laryngeal tuberculosis: use of videostroboscopy in diagnosis. Ear Nose Throat J..

